# A Novel Therapeutic Approach for Colorectal Cancer Stem Cells: Blocking the PI3K/Akt Signaling Axis With Caffeic Acid

**DOI:** 10.3389/fcell.2020.585987

**Published:** 2020-12-23

**Authors:** Se-Ra Park, Soo-Rim Kim, In-Sun Hong, Hwa-Yong Lee

**Affiliations:** ^1^Department of Health Sciences and Technology, Gachon Advanced Institute for Health Sciences & Technology (GAIHST), Gachon University, Incheon, South Korea; ^2^Department of Molecular Medicine, School of Medicine, Gachon University, Incheon, South Korea; ^3^Department of Biomedical Science, Jungwon University, Goesan-gun, South Korea

**Keywords:** colorectal cancer, cancer stem cell, caffeic acid, AKT signaling, stem-like property

## Abstract

Cancer stem cells (CSCs) have been identified in a multiple of cancer types and resistant to traditional cancer therapies such as chemotherapeutic agents and radiotherapy, which may destroy bulk tumor cells but not all CSCs, contributing to reformation tumor masses and subsequent relapse. Moreover, it is very difficult to effectively identify and eliminate CSCs because they share some common phenotypic and functional characteristics of normal stem cells. Therefore, finding better therapeutic strategies to selectively target CSCs might be helpful to reduce subsequent malignancies. In the present study, we found that caffeic acid effectively suppresses self-renewal capacity, stem-like characteristics, and migratory capacity of CD44^+^ and CD133^+^ colorectal CSCs *in vitro* and *in vivo*. In addition, we also revealed that PI3K/Akt signaling may be linked to multiple colorectal CSC-associated characteristics, such as radio-resistance, stem-like property, and tumorigenic potential. To the best of our knowledge, this is the first study demonstrating that caffeic acid effectively targets colorectal CSC populations by inhibiting the growth and/or self-renewal capacity of colorectal CSCs through PI3K/Akt signaling *in vitro* and *in vivo*.

## Introduction

The incidence and mortality rates for colorectal cancer remain considerably high in the western world and the United States (Siegel et al., 2013). Various therapeutic approaches have been developed to treat colorectal cancers, but overall mortality rate remains extremely high and unchanged (Haggar and Boushey, 2009). In fact, approximately 50% of patients with complete surgical resection will relapse and ultimately die of chemotherapy-resistant metastatic disease (Gramont, 2005; Marshall et al., 2007; Kopetz et al., 2008; De Dosso et al., 2009). Although the cause of relapse has not yet been fully elucidated, the presence of a small subset of self-renewing cancer stem cells (CSCs) within a tumor is thought to be one of the major contributing factors for tumor metastasis and recurrence (Ayob and Ramasamy, 2018; Yang et al., 2020). CSCs have indeed been identified in almost all major cancer types, including breast cancer (Al-Hajj et al., 2003), colon cancer (Ricci-Vitiani et al., 2007), and leukemia (Jamieson, 2008). Importantly, CSCs are resistant to traditional anti-cancer therapies, such as chemotherapy (Makena et al., 2020) and radiotherapy (Schulz et al., 2019). Therefore, selectively targeting and eliminating CSCs will ultimately improve outcomes for patients undergoing colorectal cancer treatments. However, a drawback is that currently available knowledge about colorectal CSCs is largely influenced by the biological features of normal stem cells. Thus, targeting these common biological characteristics to eliminate colorectal CSCs may reduce normal stem cells and subsequently prevent normal gut regeneration. In this context, special attention has been recently devoted to selectively targeting colorectal CSCs without affecting normal stem or non-stem cells.

Many investigators have attempted to effectively eliminate colorectal CSCs with various synthetic agents, such as glycosaminoglycan mimetic (Boothello et al., 2019), SN-38 nanoparticles (Alibolandi et al., 2018), curcumin analog (Su et al., 2018), checkpoint kinase 1 (Chk1) inhibitor (Manic et al., 2018). Although they achieved moderate success, the overall results were not satisfactory due to the low chemo-sensitivity of colorectal CSCs. Thus, it is hoped that a novel biologically active compound based on natural products with minimal toxicity may significantly improve the sensitivity and efficiency for targeting colorectal CSCs. Particular attention has been recently devoted to the potential inhibitory effects of caffeic acid, a main bioactive component of coffee, on multiple types of cancers, including breast (Kabala-Dzik et al., 2018), skin (Zeng et al., 2018), and liver (Espindola et al., 2019) cancers. However, the specific inhibitory effects of caffeic acid against colorectal CSCs and its underlying molecular mechanisms remain underexplored despite the fact that coffee is affordable and has a long history of human use.

In the current study, we demonstrated for the first time that caffeic acid significantly inhibited multiple colorectal CSC-associated characteristics, such as growth potential, migratory capacity, radio-resistance, pluripotency, and tumorigenicity *in vitro* and *in vivo.* More strikingly, we also demonstrate that a caffeic acid exerts anticancer activity by inhibiting the PI3K/Akt cascade *in vitro* and *in vivo*. Markedly enhanced PI3K/Akt signaling activity has recently been discovered in multiple human cancer types (Hoxhaj and Manning, 2020; Jiang et al., 2020), especially cancers of the gastrointestinal (GI) tract (Matsuoka and Yashiro, 2014; Malley and Pidgeon, 2016; Corti et al., 2019). Despite many compelling findings, many questions regarding the functional relationship between PI3K/Akt signaling activity and many of the properties of colorectal CSC properties, such as radiation-resistance, metastasis, and tumorigenesis, remain unanswered. Importantly, here, we further demonstrated that PI3K/Akt signaling, as a putative marker for colorectal CSCs, regulates intestinal tumor growth via facilitating the expression of pluripotency-related factors, migratory capability, and the resistance to radiation *in vitro* and *in vivo.* Taken together, these results suggested that inhibiting the PI3K/Akt signaling cascade with caffeic acid might be an effective therapeutic strategy that selectively targets colorectal CSCs.

## Materials and Methods

### Cell Culture and Reagents

The human colon adenocarcinoma cell line HCT116 was purchased from the Korean Cell Line Bank (KCLB, Seoul, Republic of Korea) and were cultured in Gibco^®^ RPMI 1640 medium (Invitrogen, Grand Island, NY) supplemented with 100 U/ml penicillin/streptomycin (Lonza, Basel, Switzerland) and ultracentrifuged 10% fetal bovine serum (FBS) at 37°C in a 5% CO_2_ humidified incubator. Akt activator SC79 (Cat. No: SML0749) and caffeic acid (Cat. No: C0625) were purchased from Sigma-Aldrich (St. Louis, MO). Akt inhibitor V (Cat. No: 124012) was purchased from CalBiochem (La Jolla, CA).

### Cell Proliferation Assay

The MTT assay was used to determine the cytotoxicity of caffeic acid, according to the manufacturer’s protocol. HCT116 cells (2 × 10^4^ cells/well) were seeded in 96-well plates. After 24 h of incubation, the cells were treated with an increasing concentration of caffeic acid for 48 h. The viable cells were measured at a wavelength of 570 nm using a VersaMax microplate reader.

### CSC Sphere Formation Assay

The HCT116 cell line has been widely used as an *in vitro* model of colorectal CSCs in various previous studies (Xiang et al., 2006; Chen et al., 2018; Quarni et al., 2019). Therefore, HCT116 cells were cultured in serum-free medium (Invitrogen Life Technologies, CA, United States) containing 20 ng/ml basic-fibroblast growth factor (bFGF, PeproTech Inc., Rocky Hill, NJ, United States), B27), B27 supplement minus vitamin A (Gibco, Life Technologies, 1×), 20 ng/ml epidermal growth factor (EGF, PeproTech Inc., Rocky Hill, NJ, United States), and 4 μg/ml heparin (Sigma-Aldrich, St. Louis, MO) and then plated (1 × 10^4^ cells/well) onto Corning Costar ultra-low attachment (ULA) multiwell plates. After 10 days, the formed CSC spheres from each replicate well with diameters ≥ 100 μm were counted under an inverted microscope at a magnification of 50×. The percentage of cancer cells with CSC sphere-forming ability, termed the “tumorsphere formation efficiency (TSFE),” was analyzed as follows: [(number of CSC spheres that were formed/number of single cells that were plated in multiwell plates) × 100].

### Protein Isolation and Western Blot Analysis

The protein expression levels were analyzed by western blotting as described in our previous studies (Park et al., 2020). Cells were lysed in a buffer containing 50 mM Tris, 5 mM EDTA, 150 mM NaCl, 1 mM DTT, 0.01% NP 40, and 0.2 mM PMSF. The protein concentrations of the total cell lysates were measured by using bovine serum albumin as a standard. Samples containing equal amounts of protein were separated by sodium dodecyl sulfate-polyacrylamide gel electrophoresis (SDS-PAGE) and then transferred onto polyvinylidene difluoride (PVDF) membranes (Bio-Rad Laboratories). The membranes were blocked with 5% skim milk in Tris-buffered saline containing Tween-20 at RT. Then, the membranes were incubated with primary antibodies against MMP-2 (Cell signaling #4022), MMP-9 (Cell Signaling #13667), phospho-PI3K (Cell Signaling #4228), total PI3K (Cell Signaling #4292), phospho-Akt (Cell Signaling #4060), total Akt (Cell Signaling #4491) and β-actin (Abcam, MA, United States, ab189073) overnight at 4°C and then with polyclonal HRP-conjugated goat anti-mouse IgG (BD Pharmingen, 554002) or goat anti-rabbit IgG (BD Pharmingen, San Diego, CA, United States, 554021) secondary antibodies at room temperature for 60 min. The antigen-antibody complexes were detected using Western blot ECL reagents (GE Healthcare, Bucks, United Kingdom).

### Real-Time PCR Analysis

Total RNA was extracted from monolayer cultured cells or CSC spheres using commercial TRIzol^®^ reagent (Invitrogen Life Technologies, CA, United States) according to the manufacturer’s recommended instructions. RNA purity was estimated by measuring the ratio of absorbance at 260 and 280 nm. The first-strand cDNA was synthesized using SuperScript II Reverse Transcriptase (Invitrogen Life Technologies, CA, United States) with 1 μg of total RNA. The first-strand cDNA was synthesized using Express SYBR-Green qPCR Supermix (BioPrince, Seoul, South Korea). qPCR was performed using a QIAGEN’s real-time PCR cycler, the Rotor-Gene Q. The relative mRNA expression levels of the target genes were calculated as fold changes using the ΔΔCT method. The sequences of the PCR primers are listed in Table 1.

**TABLE 1:** Primer sequences for quantitative RT-PCR.

**Gene**	**Gene bank no.**	**Direction**	**Primer sequence**
Human KLF4 NM_004235		F	TTCTCTCCAATTCGCTGACC
		R	GGATCGGATAGGTGAAGCTG
Human NANOG	NM_ 024865	F	ACATGCAACCTGAAGACGTGTG
		R	CATGGAAACCAGAACACGTGG
Human PPIA	NM_021130	F	TGCCATCGCCAAGGAGTAG
		R	TGCACAGACGGTCACTCAAA
Human SOX2	NM_003106.3	F	AAATGGGAGGGGTGCAAAAGAGGAG
		R	CAGCTGTCATTTGCTGTGGGTGATG

### Flow Cytometry

FACS analysis and cell sorting were performed using FACS Calibur and FACS Aria machines (Becton Dickinson, Palo Alto, CA), respectively. FACS data were analyzed using FlowJo software (Tree Star, Ashland, OR). Antibodies to the following proteins were used: PE-conjugated CD44 (BD Bioscience, Cat. 559942, dilution 1/40) and 133 (MACS; Miltenyi Biotech, Sunnyvale, CA, 130-080-081, dilution 1/40). The MACS MultiSort Kit (Miltenyi Biotech, 130-090-757) was used to sort for CD44^–^ CD133^–^ and CD44^+^CD133^+^ cells according to the manufacturer’s protocol. The FACS gates were established by staining with an isotype antibody or secondary antibody.

### Immunofluorescent Staining

Human colon carcinoma cells and tumor tissues were fixed with 4% paraformaldehyde (PFA) solution for 10 min at room temperature. HCT116 cells were permeabilized with 0.4 M glycine and 0.3% Triton X-100 and non-specific binding was blocked with 2% normal swine serum (DAKO, Glostrup, Denmark) as described in our previous studies (Park et al., 2020). After formalin fixation, paraffin-embedded tumor sections (4 μm) were dewaxed in xylene and rehydrated through an alcohol gradient, after which the endogenous peroxidase activity was blocked with 10% hydrogen peroxide. The tumor sections were immersed in Tris-EDTA (pH 9.0) (Thermo Fisher Scientific, Pittsburgh, PA, United States), rinsed in Tris-buffered saline and heated in a microwave oven. The samples were incubated with the following primary antibodies: anti-phospho-Akt (Cell Signaling #4060), CD44 (Abcam, ab46793), and CD133 (Biorbyt, Cambridge, United Kingdom, Orb114000, 43280) antibody. Sections were subsequently treated with secondary antibody and incubated with the avidin-biotin-peroxidase complex (MOM kit) (Vector Laboratories, Burlingame, CA, United States). The samples were examined by fluorescence microscopy (Zeiss LSM 510 Meta).

### Tumorigenesis Experiment

All of the animal experiments were approved and carried out in accordance with the Institutional Animal Care and Use Committee (IACUC) (LCDI-2013-0031) of the Lee Gil Ya Cancer and Diabetes Institute of Gachon University. To evaluate the efficacy of caffeic acid, we used xenograft animal models. HCT116 cells (3 × 10^6^ cells/mouse) were injected into the left flank of NSG mice. After tumor inoculation, the tumor volume reached approximately 100 mm^3^, and the mice were randomly divided into control (vehicle) and caffeic acid-treatment (10 mg/kg, I.P., daily) groups. The tumor size was measured twice a week, and the tumor volume was calculated with following formula: volume = (longitudinal × transverse^2^)/2. Additionally, the 3D tumor volume was calculated using a volumetric micro-CT scanner (NFR-Polaris G90, NanoFocusRay, Iksan, South Korea) before necropsy.

### Patient-Derived Tumor Xenograft (PDTX) Model

Human tumor samples were obtained from patients diagnosed with colorectal cancer, after written informed consent was obtained from all patients. All research was approved by the Institutional Review Board of Gachon University College of Medicine (GCIRB-2013-66). Fresh tumor tissues were maintained in DMEM (Invitrogen) supplemented with 10% fetal bovine serum (FBS), 1% cefotiam hydrochloride (Hanmi Pharma, Seoul, Korea), 100 U/ml penicillin, and 100 U/ml streptomycin (Lonza). To establish the PDTX models, fresh tumor specimens were collected immediately after surgery and rinsed with antibiotic-containing DMEM medium. The tumor tissues were minced into small pieces (3 × 3 × 3 mm) and implanted subcutaneously into NSG mice. Six-to eight-week-old female NSG mice (NOD.Cg-Prkdc scid Il2rg tm1Wjl/SzJ, Jackson Laboratory, Bar Harbor, ME, United States) were used for the implantation of tumor tissues. After the tumor grew to a mean diameter of 10 mm or reached a tumor volume of 1,000 mm^3^, the mice were sacrifice, and the tumor tissues were serially passaged into additional mice.

### Transwell Migration Assay

Colorectal cancer cells were seeded at a density of 1 × 10^5^ cells/well in BD Falcon polycarbonate transwell inserts (8 μm pore size membranes for 24 well/plate) to track the migration capacity of plated cells. Non-migrating cells on the upper surface of polycarbonate membranes of transwell inserts were removed by scrubbing with a cotton tipped swab as described in our previous studies (Park et al., 2019). Migrated cells on the lower surface of polycarbonate membranes of transwell inserts were fixed with 4% paraformaldehyde (PFA) for 10 min and then stained with hematoxylin for 20 min at room temperature. Next, the number of migratory cells were analyzed and counted in three randomly selected fields per polycarbonate membrane from each condition under a light microscope.

### Oncomine Database Analysis

We used the Oncomine Cancer Microarray database^1^ to analyze the activity of Akt signaling between relapse-free and relapse colorectal cancer patients or non-metastatic and metastatic colorectal cancer patients (Seiber cohort dataset). The expression value of Akt was log2 transformed followed by median centering for each gene. All statistical analyses were performed with GraphPad Prism 5.0 (GraphPad Software, Inc., La Jolla, CA, United States).

### Gene Set Enrichment Analysis (GSEA) of Various Recurrent and Metastatic Colorectal Cancers

Clinical patient data from multiple types of colorectal cancers were collected and analyzed using the Seiber cohort dataset (GSE14333) (Jorissen et al., 2009) from “R2: Genomics Analysis and Visualization Platform^2^.” The primary data are available from GEO^3^ under series accession no. GSE143335. GSEA to identify pathways enriched in the ranked gene lists filtered by a particular threshold was performed using the Java implementation of GSEA obtained from http://www.broadinstitute.org/gsea/. Differentially expressed genes (DEGs) between non-metastatic and metastatic or between non-recurrent and recurrent colorectal cancer were analyzed based on the relative fold-change obtained from the Seiber cohort dataset (GSE14333). The analysis included gene sets from MSigDB pathways, C2: all curated genes (c2.all.v5.0.symbols.gmt) or c6: oncogenic signature gene sets (c6.all.v5.0.symbols.gmt). The normalized enrichment score (NES) accounts for differences in gene set size. The FDR *q*-value (the probability that a gene set with a given NES represents a false-positive finding) was used to set a significance threshold.

### Ingenuity Upstream Regulator Analysis

An “upstream regulator” analysis was performed with Ingenuity Pathway Analysis (IPA) version 2.0 software^4^ (Ingenuity Systems, Inc., Redwood City, CA). Differential gene expression profiles (*t*-test, *P* < 0.05) between non-metastatic and metastatic colon cancer were subjected to the subsequent upstream regulator analysis. The statistical significance of target signaling regulator was analyzed by Fisher’s exact test, which was used to determine differentially expressing genes from the microarray results. The activation score (*z* score) was used to predict the activation/inhibition status of target molecules by comparing the identified differentially regulated genes (either consistently up-regulated or consistently down-regulated) in the dataset.

### Statistical Analysis

All experiment data were represented as the mean ± *SD* and were based on at least three different experiments. The statistical analyses among the experimental groups were carried out using GraphPad Prism 5.0 (GraphPad Software, Inc., La Jolla, CA, United States) following one-way ANOVA. A *p*-value of less than 0.05 can be considered as a significant result.

## Results

### Caffeic Acid Effectively Suppresses Self-Renewal Capacities and Stem-Like Properties of Colorectal CSCs *in vitro*

**FIGURE 1:**
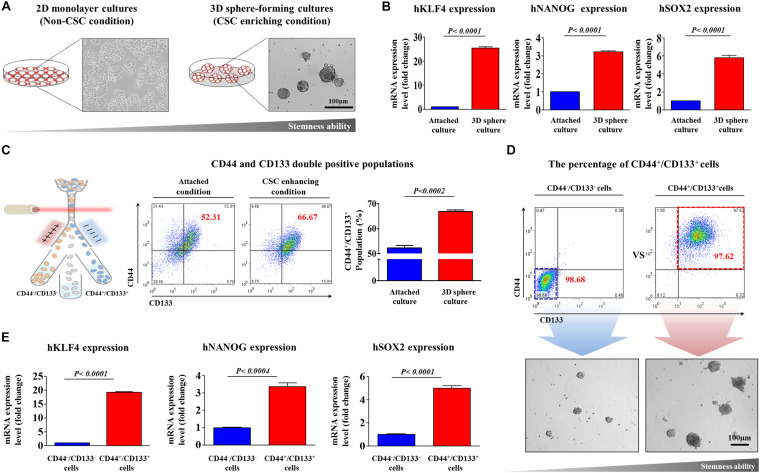
Establishment of a 3D-sphere-forming culture system as an *in vitro* culture model of colorectal CSCs. Colorectal CSC spheres were formed using the HCT116 cancer cell line after 1 week sphere culture. The sizes of spheres greater than 100 μm were enumerated, with a representative image of a tumorsphere shown **(A)**. Real-time PCR results demonstrating changes in the expression of the stem cell markers KLFG, NANOG, and SOX2 after 1 week in sphere culture relative to that in subconfluent monolayers **(B)**. The results of FACS analysis showing the percentage of the total cell population that consisted of CD44^+^/CD133^+^ cells in both monolayer and sphere cultures **(C)**. HCT116 cells were sorted by dual-color flow cytometry analysis according to CD44 and CD133 expression. The dot plot is divided into two quadrants for CD44^+^/CD133^+^ or CD44^–^/CD133^–^. The sorted HCT116 cell populations were plated into sphere-forming culture dishes, and their clonogenic abilities were analyzed **(D)**. Real-time PCR results demonstrating changes in the expression of KLFG, NANOG, and SOX2 in both CD44^+^/CD133^+^ and CD44^–^/CD133^–^ subpopulations **(E)**. The results represent the means ± *SD* of three independent experiments.

**FIGURE 2:**
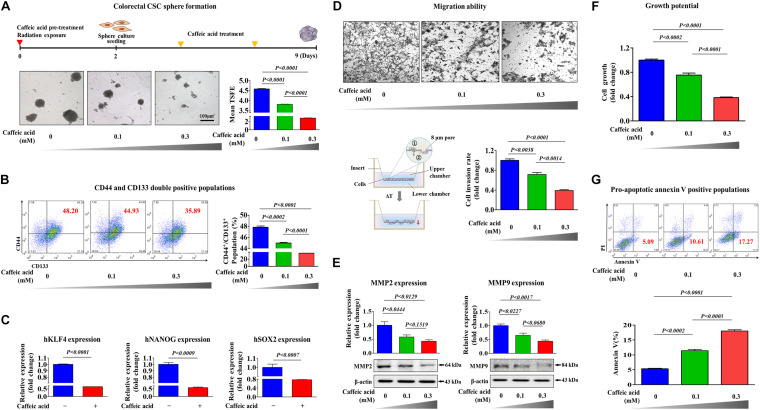
The inhibitory effects of caffeic acid on the clonogenicity and pluripotency of colorectal CSCs. Colorectal CSCs are more resistant to radiation than bulk tumor cells. Therefore, primary CSC spheres were exposed to radiation (2Gy) and dissociated into single cells. These cells were subsequently replated onto culture dishes without additional radiation exposure to form secondary CSC spheres. Caffeic acid treatment inhibited sphere formation of colorectal CSCs **(A)**. The percentage of CD44^+^ CD133^+^ subpopulations was evaluated by FACS analysis. The treatment of HCT116 cells with caffeic acid (0.3 mM) for 48 h decreased the percentage of CD44^+^ CD133^+^ cells in the total cell population **(B)**. Real-time PCR results reveal the changes in the expression of KLFG, NANOG, and SOX2 in both the vehicle-and caffeic acid-treated groups **(C)**. HCT116 cells were treated with caffeic acid for 24 h, after which the effect of caffeic acid on cell migration ability was evaluated using a transwell migration assay **(D)**. The relative expression levels of key positive regulators of cell migration (MMP-2/9) were assessed using western blotting **(E)**. The inhibition of cell viability by caffeic acid treatment for 72 h was determined by an MTT assay. Cell viability (%) was calculated as a percent of the vehicle control **(F)**. Caffeic acid-induced cytotoxicity was evaluated by flow cytometry using PE-labeled Annexin-V **(G)**. β-actin was used as the internal control. The data represent the mean ± *SD* of three independent experiments.

To determine whether caffeic acid inhibits the tumorigenicity and various stem-cell-like properties of colorectal CSCs, we first established a 3D-sphere-forming culture system as an *in vitro* culture model of colorectal CSCs. Previous studies have revealed that stem cell-like characteristics are effectively enriched in non-adhesive sphere culture system in multiple types of cancers, including breast (Lee et al., 2019), colon (Olejniczak et al., 2018), lung (Zhang et al., 2018), and liver (Ma et al., 2019) cancers. As expected, the expression levels of pluripotency-related related factors, such as KLF4, NANOG, and SOX2, were more highly expressed in spheroid-forming cells than in the adhesive cells (Figures 1A,B). Previous studies have suggested that CD44 (Du et al., 2008; Guo and Frenette, 2014) and CD133 (Kazama et al., 2018) are prognostic markers of poor survival and responses. Therefore, to further analyze the clonogenic potential in these putative CSC-marker positive cells, CD44^–^ CD133^–^ and CD44^+^ CD133^+^ cells were sorted by dual-color flow cytometry (Figure 1C). The double-positive cells showed significantly higher clonogenic potential than the double-negative cells (Figure 1D). We then further analyzed the expression levels of multiple pluripotency-related related genes in these putative CSC-marker positive cells; CD44^–^ CD133^–^ and CD44^+^ CD133^+^ cells were sorted by dual-color flow cytometry. Consistently, the expression levels of multiple pluripotency-related related factors were significantly higher in double-positive cells than in the double-negative cells (Figure 1E). Next, we tested the efficacy of caffeic acid to suppress the self-renewal capacities and stem-like properties of colorectal CSCs. Importantly, normal human fibroblast cell-based dose-dependent experiments showed no marked signs of toxicity at the caffeic acid dose used in this study (Supplementary Figures 1A,B). More strikingly, the clonogenic potential (Figure 2A) and proportion of CD44^+^ CD133^+^ cells (Figure 2B) were significantly suppressed by caffeic acid treatment, in a dose dependent manner. Consistent with these finding, the relative expression levels of pluripotency-related related factors were also significantly decreased in the caffeic acid-treated colorectal CSCs (Figure 2C). We further assessed the effects of caffeic acid on the migratory capacity of colorectal CSCs using a transwell invasion assay. The ability to migrate across the transwell membrane (Figure 2D) and the expression of matrix metalloproteinase 2/9 (MMP-2/9), which plays a crucial role in regulating cell migration and tissue regeneration (Figure 2E), were markedly decreased in caffeic acid-treated cells compared to non-treated cells. Previous studies have revealed that reorganization of the actin cytoskeleton is associated with cell migration by pushing or pulling on the plasma membrane (Svitkina, 2018). Consistently, phalloidin staining of actin cytoskeleton revealed a significant correlation between caffeic acid treatment and dynamic actin filament rearrangement (Supplementary Figure 2), suggesting that the markedly suppressed migratory ability of caffeic acid-treated cells may be associated with actin filament rearrangement. Moreover, these inhibitory effects of caffeic acid on tumorigenicity may be related to the growth potential and apoptotic processes, including the Annexin V-positive population (Figures 2F,G). Additionally, elevated pro-apoptotic DNA fragmentation was also observed with caffeic acid-treated cells (Supplementary Figure 3).

### Caffeic Acid Effectively Suppresses Radio-Resistant Colorectal CSCs *in vitro*

**FIGURE 3:**
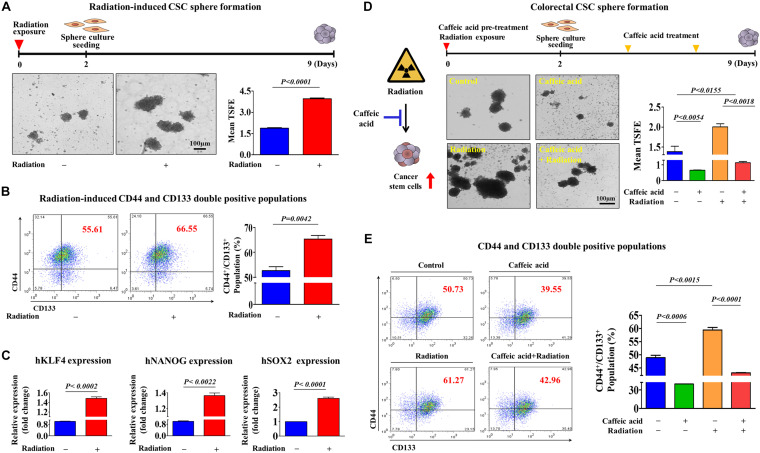
Caffeic acid effectively suppresses radiotherapy-induced clonogenicity and pluripotency of colorectal CSCs. Colorectal CSC spheres derived from HCT116 cells were exposed to radiation (2Gy). Radiation significantly increased CSC sphere formation **(A)**. The percentages of CD44^+^ CD133^+^ subpopulations were evaluated by FACS analysis. The exposure of HCT116 cells to radiation (2Gy) increased the percentage of CD44^+^ CD133^+^ cells in the total cell population **(B)**. The mRNA levels of KLFG, NANOG, and SOX2 in both control and radiation (2Gy)-exposed cells were measured using real-time PCR **(C)**. Primary CSC spheres were exposed to radiation (2Gy) and dissociated into single cells. These cells were subsequently replated onto culture dishes with or without additional caffeic acid (0.3 mM) treatment to form secondary CSC spheres. Caffeic acid inhibited radiation-resistant sphere formation in HCT116 cells **(D)**. The treatment of HCT116 cells with caffeic acid for 48 h decreased the percentage of radiation-induced CD44^+^ CD133^+^ cells in the total cell population **(E)**. The sphere sizes greater than 100 μm were enumerated, and a representative image of a tumor sphere is shown. Abbreviations: TSFE, tumor sphere-forming efficiency. The results are presented as the means ± *SD* of three independent experiments.

It has been suggested that colorectal CSCs are associated with resistance to various conventional therapeutic approaches, including chemotherapy (Phi et al., 2018) and radiotherapy (Arnold et al., 2020). Consistent with these studies, we observed that both the size of sphere formation (Figure 3A) and the percentage of CD44/CD133 double positive populations (Figure 3B) in colorectal CSCs were markedly increased in response to 2Gy radiation. Furthermore, we also found that the expression levels of pluripotency-related related factors, such as KLF4, NANOG, and SOX2, were also significantly increased following 2Gy radiation (Figure 3C). Importantly, radiation-enriched colorectal CSC sphere formation (Figure 3D) as well as the percentages of CD44/CD133 double positive populations (Figure 3E) were significantly reduced by caffeic acid exposure. These results indicate that caffeic acid effectively targets colorectal CSC populations, which can be enriched by conventional chemotherapeutic agents and radiotherapy.

### Aberrant Activation of Akt Signaling Is Linked to Multiple CSC-Associated Characteristics

**FIGURE 4:**
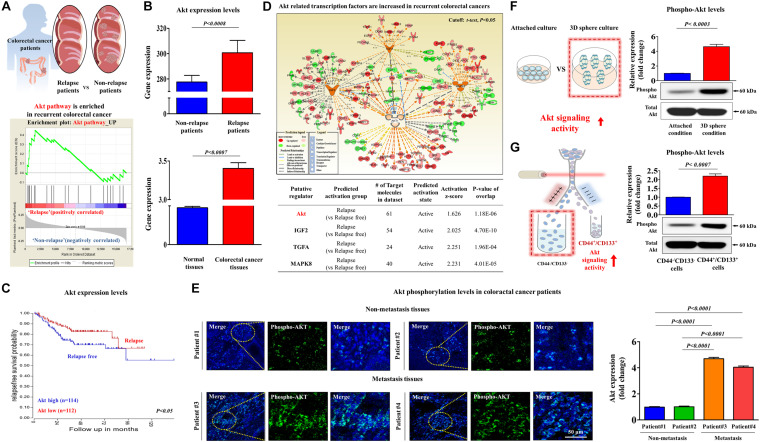
Aberrant activation of Akt signaling is associated with metastatic progression in colorectal cancer. Differentially expressed genes from metastatic and non-metastatic colorectal cancers (Seiber cohort, GSE14333) were applied to a gene set enrichment analysis (GSEA). The GSEA revealed the highly enhanced expression of Akt signaling in recurrent colorectal cancer **(A)**. The available colorectal cancer datasets were analyzed using the Oncomine dataset repository (www.oncomine.org). After specifically filtering for colorectal cancer datasets showing a frequency of tumor recurrence, we found significant correlations between enhanced Akt signaling and a higher overall recurrence **(B)** as well as a lower survival rate **(C)**. An upstream regulator analysis was performed with ingenuity pathway analysis (IPA) software (http://www.ingenuity.com) to predict the activation state (either activated or inhibited) of several Akt-dependent putative regulators in recurrent vs. non-recurrent cases. The “Z-score” profile indicates whether a specific putative regulator is significantly more “activated” than “inhibited.” The IPA analysis revealed the highly enhanced expression of Akt signaling-related transcription factors, such as Akt, IGF-2, TGF-α, and MAPK8, in recurrence **(D)**. Non-metastatic and metastatic colorectal cancer tissues were stained with an antibody specific for phospho-Akt. DAPI staining was used to label the nuclei within each field **(E)**. Western blotting results demonstrate the changes in the activity of Akt signaling after 1 week in sphere culture relative to that in subconfluent monolayers **(F)**. HCT116 cells were sorted by dual-color flow cytometry analysis according to CD44 and CD133 expression. The dot plot is divided into two quadrants for CD44^+^ CD133^+^ or CD44^–^ CD133^–^. Western blotting results demonstrate the changes in the activity of Akt signaling in both CD44^+^CD133^+^ and CD44^–^ CD133^–^ subpopulations **(G)**. β-actin was used as the internal control. The data represent the mean ± *SD* of three independent experiments.

It has been recently suggested that colorectal CSCs contribute to tumor recurrence and metastasis (Prager et al., 2019). Therefore, to identify potential target genes that are significantly activated in metastatic progression or recurrent patients, we performed a gene set enrichment analysis (GSEA), which is an algorithm for determining whether the differentially expressed genes are enriched for particular physiological conditions, on clinical data of colorectal cancer with the Seiber dataset (GSEA14333). Interestingly, the GSEA results revealed that Akt signaling components were markedly enriched in recurrent colorectal cancer (Figure 4A). Furthermore, we performed a correlation analysis to verify the potential pathological link between Akt signaling and recurrence of colorectal cancer using the Oncomine dataset repository^5^. The gene datasets were filtered by Akt signaling activity and occurrence or recurrence of colorectal cancer. The results revealed a strong relationship between markedly enhanced Akt signaling activity and the occurrence or recurrence of colorectal cancer (Figure 4B). We also observed significant correlations between enhanced Akt signaling activity and lower survival rates or higher rates of recurrence (Figure 4C). Furthermore, to assess the activation state of these Akt signaling components in recurrent vs. non-recurrent colorectal cancers, we examined the gene expression profiles using ingenuity pathway analysis (IPA) software. Consistently, positive regulators of Akt signaling, such as Akt (*Z*-score = 1.626, *p* = 1.18E–06), IGF2 (Z-score = 2.025, *p* = 4.70E–10), TGFA (Z-score = 2.251, *p* = 1.96E–04), and MAPK8 (Z-score = 2.231, *p* = 4.01E–05), were activated in recurrent colorectal cancers (Figure 4D). Consistent with the results of clinical big data analytics, our immunostaining revealed that the expressions level of phospho-Akt was significantly enhanced in recurrent tumor tissues compared with that in non-recurrent tumor tissues (Figure 4E). To further investigate the correlations between Akt signaling activity and colorectal CSC subpopulations, we analyzed the expression levels of phospho-Akt in spheroid-forming CSCs (Figure 4F) or in CD44^+^ CD133^+^ double-positive cells (Figure 4G). These results suggested that aberrant activation of Akt signaling may be linked to multiple CSC-associated characteristics, such as radio-resistance, stem-like property, and tumorigenic potential of colorectal CSCs.

### Inhibitory Effects of Caffeic Acid on Colorectal CSCs Are Achieved by Disrupting the PI3K/Akt Signaling Cascade

**FIGURE 5:**
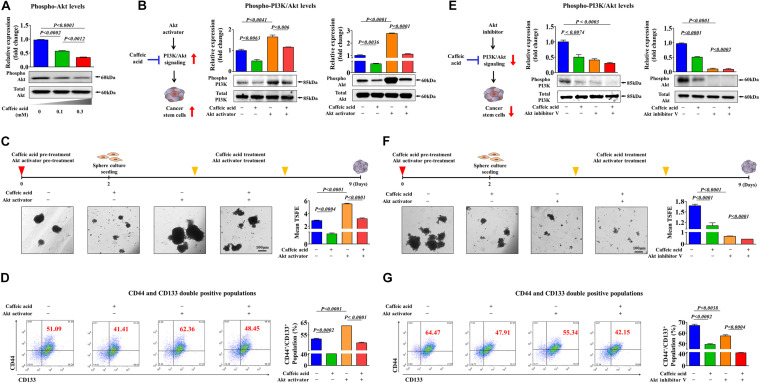
Activation or inhibition of Akt signaling modulates the caffeic acid-induced effects on clonogenicity and pluripotency of colorectal CSCs. Western blotting results reveal the changes in Akt activity in both vehicle- and caffeic acid-treated groups **(A)**. Colorectal CSCs were pretreated with the Akt activator SC79 (10 μM) for 24 h prior to treatment with 0.3 mM caffeic acid for 48 h, and the changes in the activities of PI3K and Akt were determined by western blotting **(B)**. The attenuating effects of Akt activation on the caffeic acid-induced sphere-forming ability and the percentage of CD44^+^CD133^+^ subpopulations were assessed by sphere-forming culture **(C)** and FACS analysis **(D)**, respectively. Colorectal CSCs were pretreated with the Akt inhibitor V (10 μM) for 24 h prior to treatment with 0.3 mM caffeic acid for 48 h, and the changes in the activities of PI3K and Akt were determined by western blotting **(E)**. The synergistic effects of Akt inhibition on the caffeic acid-induced sphere-forming ability and the percentage of CD44^+^ CD133^+^ subpopulations were assessed by sphere-forming culture **(F)** and FACS analysis **(G)**, respectively. The sphere sizes greater than 100 μm were enumerated, and a representative image of a tumor sphere is shown. Abbreviations: TSFE, tumor sphere-forming efficiency. β-actin was used as the internal control. The data represent the mean ± *SD* of three independent experiments.

To investigate the underlying molecular mechanisms of the caffeic acid-induced inhibitory effects, we evaluated whether caffeic treatment was sufficient to activate the PI3K/Akt signaling cascade in colorectal CSCs. Importantly, the phosphorylation levels of Akt were significantly decreased in caffeic acid-treated cells in a dose dependent manner (Figure 5A). Therefore, it is reasonable to hypothesize that caffeic acid inhibits the self-renewal and stem cell-like properties of colorectal CSCs by disrupting the Akt signaling axis. In this context, to confirm whether Akt signaling can mediate caffeic acid-induced effects on colorectal CSCs, we activated or inhibited Akt signaling using the specific Akt activator SC79 or the Akt inhibitor V, respectively. Indeed, the Akt activator-induced stimulatory effects of PI3K/Akt signaling were significantly attenuated by treatment with caffeic acid (Figure 5B). Importantly, the inhibitory effects of caffeic acid on colorectal CSC-sphere formation (Figure 5C) and the percentage of the total cell population that consisted of CD44^+^ and CD133^+^ cells (Figure 5D) were successfully attenuated by Akt signaling activation. In addition, the Akt inhibitor-induced inhibitory effects of PI3K/Akt signaling were markedly synergized by treatment with caffeic acid (Figure 5E). Consistent with the Akt signaling activation results, the inhibitory effects of caffeic acid on colorectal CSC-sphere formation (Figure 5F) and the percentage of the total cell population that consisted of CD44^+^ and CD133^+^ cells (Figure 5G) were also synergized by Akt signaling inhibition. These results suggested that the inhibitory effects of caffeic acid on the self-renewal and stem-like properties of colorectal CSCs may be achieved by disrupting the PI3K/Akt signaling axis.

### Caffeic Acid Suppresses Radiation-Induced Self-Renewal and Stem-Like Properties of PDTX-Derived Colorectal CSCs

**FIGURE 6:**
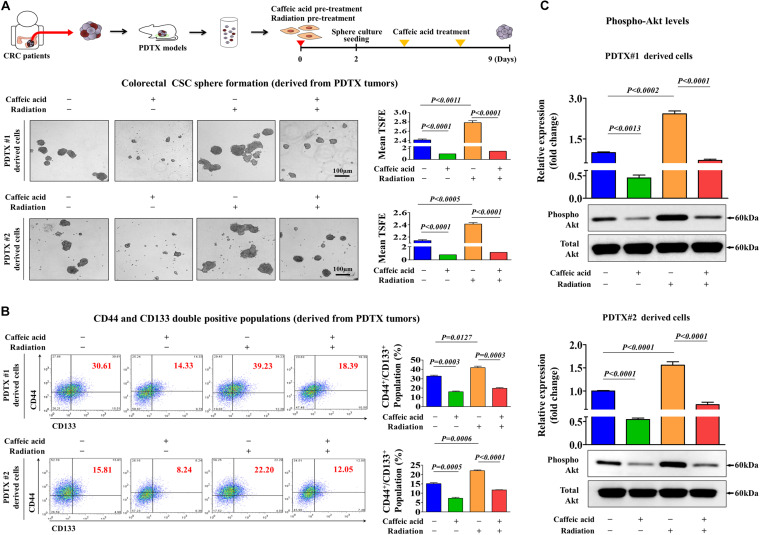
Caffeic acid suppresses the clonogenicity and pluripotency of colorectal CSCs by disrupting the Akt signaling axis in the PDTX model. Schematic representation of the experimental protocol as described in the section “Materials and Methods.” Anesthetized 7 weeks old male NSG mice were inoculated with a 1:1 mixture of Matrigel and 3 × 10^6^ HCT116 cells into the subcutaneous tissue. Next, primary CSC spheres derived from two PDTX mice were exposed to radiation (2Gy) and dissociated into single cells. These cells were subsequently replated onto culture dishes without additional caffeic acid exposure to form secondary CSC spheres. Caffeic acid treatment successfully attenuated radiation-induced sphere formation of colorectal CSCs **(A)**. The percentage of CD44^+^CD133^+^ subpopulations was evaluated by FACS analysis. The treatment of HCT116 cells with caffeic acid (0.3 mM) for 48 h decreased the percentage of CD44^+^ CD133^+^ cells in the total cell population **(B)**. Colorectal CSC spheres derived from two PDTX mice were exposed to radiation (2Gy) with or without additional caffeic acid exposure and the activities of Akt signaling were determined by western blotting **(C)**. The sphere sizes greater than 100 μm were enumerated, and a representative image of a tumor sphere is shown. Abbreviations: TSFE, tumor sphere-forming efficiency. β-actin was used as the internal control. The data represent the mean ± *SD* of three independent experiments.

A patient-derived tumor xenograft (PDTX) model of colorectal cancer can be created by directly implanting cancer tissues obtained from colorectal cancer patients into immunodeficient mice (Inoue et al., 2019; Yoshida, 2020). Therefore, the PDTX model is a promising model to solve the discrepancy of the positive therapeutic effect observed in *in vitro* cultured cancer cell lines that have adapted to growth outside the natural tumor microenvironment but the lack of an effect observed in heterogeneous tumors from patients. Here, to further evaluate the potency and efficiency of caffeic acid, we successfully established a PDTX model of human colorectal cancer. Importantly, radiation-induced CSC sphere formation (Figure 6A) and CD133^+^ and CD44^+^ cells (Figure 6B) were significantly attenuated by the caffeic acid treatment in two PDTX models. Importantly, the phosphorylation levels of Akt were significantly increased by radiation, and these radiation-induced stimulatory effects of PI3K/Akt signaling were significantly attenuated by treatment with caffeic acid (Figure 6C) in two PDTX models. These results suggested that radiation-related resistance and stem cell-like properties of colorectal CSCs may be disrupted by caffeic acid exposure.

### Caffeic Acid Effectively Suppresses Self-Renewal Capacities and Stem-Like Properties of Colorectal CSCs *in vivo*

**FIGURE 7:**
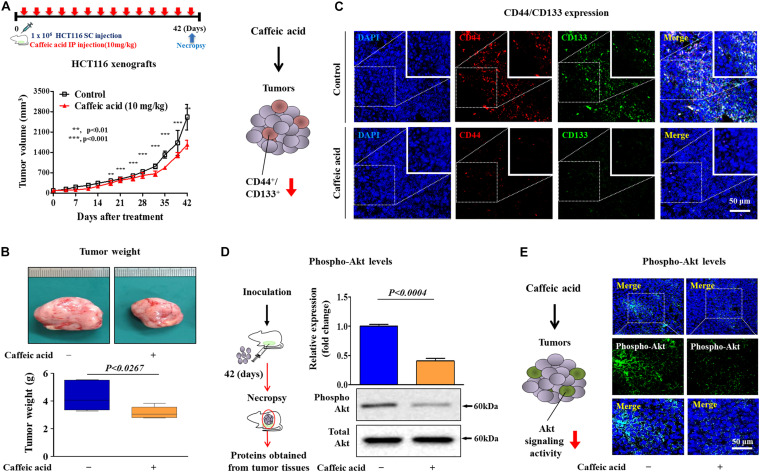
Caffeic acid suppresses the clonogenicity and pluripotency of colorectal CSCs by disrupting Akt signaling in a mice xenograft model. Schematic representation of the experimental protocol as described in the section “Materials and Methods.” Anesthetized 7 weeks old male NSG mice were inoculated with a 1:1 mixture of Matrigel and 3 × 10^6^ HCT116 cells into the subcutaneous tissue. Mice bearing HCT116 cell tumors were treated with caffeic acid (10 mg/kg, intraperitoneally) or vehicle (PBS). The tumor volumes **(A)** and weights **(B)** were measured as described in the section “Materials and Methods.” Non-treated and caffeic acid-treated colorectal cancer tissues were stained with antibodies specific for CD44 and CD133. DAPI staining was used to label the nuclei within each field **(C)**. The relative activation levels of Akt signaling in the HT29 cell xenograft models were assessed by western blotting **(D)**. Non-treated and caffeic acid-treated colorectal cancer tissues were stained with an antibody specific for phospho-Akt. DAPI staining was used to label the nuclei within each field **(E)**. β-actin was used as the internal control. The data represent the mean ± *SD* of three independent experiments.

Following our *in vitro* experiments, we further investigated the *in vivo* efficacy of caffeic acid on tumorigenesis in human tumor xenografts. Consistent with the *in vitro* results, there was a significant reduction in tumor volume (Figure 7A) and weight (Figure 7B) in mice that were treated with caffeic acid (10 mg/kg, administered intraperitoneally), suggesting that caffeic acid treatment markedly impaired the tumor-initiating potential of colorectal CSCs. To further determine whether caffeic acid treatment affects the stem cell-like properties of colorectal CSCs *in vivo*, we assessed the percentage of the CD44^+^ CD133^+^ subpopulation of tumor-xenograft-derived cells. Consistently, caffeic acid treatment successfully suppressed the percentage of the CD44^+^/CD133^+^ subpopulation *in vivo* in the xenograft model (Figure 7C). Then, to determine whether caffeic acid successfully inhibits the Akt signaling pathway *in vivo*, we investigated the expression levels of phospho-Akt in mice with or without caffeic acid treatment. As expected, both western blotting and immunohistochemistry results showed that caffeic acid treatment led to a significant decrease in phospho-Akt levels in human tumor xenograft (Figures 7D,E). These results suggested that the inhibitory effects of caffeic acid on the self-renewal and stem-like properties of CSCs *in vivo* may be achieved by disrupting the Akt signaling pathway, thus suppressing the tumorigenicity of colorectal CSCs.

## Discussion

It is now widely accepted that natural resource-based products make significant contributions to drug development and public health in terms of the treatment and/or prevention of various diseases, including cancers (Ko and Moon, 2015; Wali et al., 2019). Currently, this strategy has proven very successful for many therapeutic agents or drug candidates that are derived from plant, microbial, and semi-synthetic compounds based on natural product templates (Cheuka et al., 2016). It is commonly known that more than 80% of pharmaceutical products, including various anti-tumor agents, are obtained from natural products, such as camptothecin from *Camptotheca acuminate* (Schaeppi et al., 1974; Gunasekera et al., 1979), epipodophyllotoxins from *Podophyllum peltatum* (Krishan et al., 1975; Muggia and Gill, 1989), taxanes from *Taxus brevifolia* (Kingston et al., 1982), vinca alkaloids from *Vinca major* (Jordan et al., 1991), and so on. The therapeutic effects of numerous natural product-derived drugs have been evaluated for the management and prevention of many different types of cancer (Aung et al., 2017; Mitra and Dash, 2018). The major advantages of using natural resource-based products particularly for cancer treatment are as follows: (a) minimal or low adverse effect; (b) their ability to react to many cancer types; (c) comparably easy accessibility; and (d) the mixture of multiple potent anti-tumor components (Lee et al., 2016). Among various natural substances, caffeic acid has been reported to possess anti-inflammatory (Dos Santos and Monte-Alto-Costa, 2013), antioxidant (Sulaiman et al., 2014), and anti-tumor effects (Lin et al., 2015). Indeed, Wang et al. (2005) revealed that caffeic acid significantly induced G0/G1 phase cell cycle arrest and apoptotic cell death by suppressing the Wnt/β-catenin signaling pathway in colorectal cancer cell line HCT-116. Xiang et al. (2006) also found that caffeic acid reduced the self-renewal capacities of various colocrectal cancer cells, such as HCT-116 and SW480 cells by inhibiting cyclin D1 and c-myc signaling cascades. In this context, we investigated the therapeutic effect of caffeic acid on colorectal CSCs, which are thought to be a major contributing factor to tumor metastasis and recurrence (Dean et al., 2005). Indeed, caffeic acid effectively suppressed self-renewal capacities and stem-like properties of colorectal CSCs *in vitro* (Figures 2A–G) and *in vivo* (Figures 7A–E). Importantly, at the dose used here, caffeic acid showed no marked signs of toxicity in normal human fibroblast cell-based dose-dependent experiments (Supplementary Figure 1). Caffeic acid also effectively suppressed radio-resistant colorectal CSCs *in vitro* (Figures 3A–E). These results indicate that caffeic acid effectively targets colorectal CSC populations, which can be enriched by conventional chemotherapeutic agents and radiotherapy with minimal toxicity.

Recent studies have revealed that the PI3K/Akt signaling pathway is frequently dysregulated in colorectal cancer (Slattery et al., 2018; Zhong et al., 2019). Therefore, aberrant PI3K/Akt signaling might be an important clinical characteristic of colorectal cancers and a prognostic marker of the overall survival rate (Malinowsky et al., 2014). Consistently, our data revealed that PI3K/Akt signaling was significantly higher in metastatic colorectal cancers than in non-metastatic cancer, although both cancers exhibit a basal level of PI3K/Akt signaling (Figures 4A–E). Furthermore, PI3K/Akt signaling was significantly higher in colorectal CSCs than in bulk cancer cells (Figures 4F,G). These results suggested that targeting colorectal CSCs with a PI3K/Akt signaling inhibitor might be a more effective therapeutic strategy. Although recently developed PI3K/Akt signaling inhibitors, such as MK-2206 (Hirai et al., 2010), Perifosine (Bendell et al., 2011), and Afuresertib (Tolcher et al., 2015), effectively inhibit PI3K/Akt signaling under *in vitro* conditions, the poor activities or toxicities of these inhibitors have prevented their clinical applications (Mundi et al., 2016). Therefore, the development of novel active compounds that effectively target PI3K/Akt signaling implicated in colorectal CSC-mediated tumorigenesis and resistance is urgently needed. In addition, our results from gene set enrichment analysis (GSEA) (Figure 4B), Oncomine dataset repository (Figure 4C), ingenuity pathway analysis (IPA) (Figure 4D) results showed that the expression levels and signaling activities of Akt were markedly enriched in recurrent colorectal cancer. Interestingly, our western blotting results revealed that the expression levels of total Akt were not changed in more tumorigenic spheroid-forming CSCs (Figure 4F) or in CD44^+^ CD133^+^ double-positive cells. Although our hypothesis is that Akt expression and its signaling activity are increased during the development and metastasis/recurrence of colorectal cancer, total Akt levels were not changed in more tumorigenic spheroid-forming CSCs due to the following limitation of our *in vitro* culture model of colorectal CSCs: our *in vitro* models of colorectal CSCs are 3D-sphere-forming culture system or sorted cell culture system with putative CSC-marker (CD44 and CD133) of same colon cancer cell line (HCT116), whose expression levels of total Akt were not altered during 3D culture or cell sorting.

To further investigate whether caffeic acid effectively inhibits the growth and/or self-renewal capacity of colorectal CSCs through the suppression of PI3K/Akt signaling, we cotreated colorectal CSCs with caffeic acid and the Akt activator SC79 or the Akt inhibitor V, respectively. Importantly, the inhibitory effects of caffeic acid on colorectal CSC-sphere formation (Figure 5C) and the percentage of the total cell population that consisted of CD44^+^ and CD133^+^ cells (Figure 5D) were successfully attenuated by Akt signaling activation. The observation of markedly synergized inhibitory effects on colorectal CSC-sphere formation cotreated with caffeic acid and the Akt inhibitor further supports this interpretation (Figures 5F,G). These results suggested that the inhibitory effects of caffeic acid on the self-renewal and stem-like properties of colorectal CSCs may be achieved by disrupting the PI3K/Akt signaling axis.

Taken together, our findings suggest that the PI3K/Akt signaling cascade may facilitate self-renewal and radio-resistance and that this signaling axis may serve as a promising prognostic marker and promising therapeutic target for colorectal CSCs. To the best of our knowledge, this is the first study demonstrating the caffeic acid effectively targets colorectal CSC populations by inhibiting the growth and/or self-renewal capacity of colorectal CSCs through PI3K/Akt signaling *in vitro* and *in vivo*.

## Data Availability Statement

The original contributions presented in the study are included in the article/Supplementary Material, further inquiries can be directed to the corresponding author/s.

## Ethics Statement

The studies involving human participants were reviewed and approved by the Institutional Review Board of Gil Hospital of Gachon University (GCIRB-2013-66). The patients/participants provided their written informed consent to participate in this study. The animal study was reviewed and approved by Institutional Review Board of Gil Hospital of Gachon University (GCIRB-2013-66).

## Author Contributions

H-YL and I-SH: conception and design. S-RP and S-RK: development of methodology and performance of experiments. S-RP, S-RK, H-YL, and I-SH: analysis and interpretation of data. S-RP, H-YL, and I-SH: writing the manuscript. All authors contributed to the article and approved the submitted version.

## Conflict of Interest

The authors declare that the research was conducted in the absence of any commercial or financial relationships that could be construed as a potential conflict of interest.

## Footnotes

1 http://www.oncomine.org/

2 http://r2.amc.ml

3 http://www.ncbi.nlm.nih.gov/geo/

4 https://analysis.ingenuity.com

5 www.oncomine.org

## Supplementary Material

Supplementary Figure 1 IC_50_, the concentration that inhibits 50% of the proliferation of human dermal fibroblasts and HCT116 cells. The inhibition of cell viability by caffeic acid treatment for 48 h was determined by an MTT assay in human dermal fibroblasts **(A)** and HCT116 **(B)** cells. Cell viability (%) was calculated as a percent of the vehicle control. The results are presented as the mean ± *SD* of three independent experiments.

Supplementary Figure 2 The inhibitory effects of caffeic acid on the migratory capacity of HCT116 cells. Caffeic acid-induced actin filament disorganization and the morphological transition of HCT116 cells were visualized by staining the actin filaments with phalloidin. DAPI staining was used to label the nuclei within each field.

Supplementary Figure 3 The stimulatory effects of caffeic acid on the apoptotic DNA fragmentation in HCT116 cells. Caffeic acid-induced apoptotic DNA fragmentation and condensation were visualized using DAPI staining. The results are presented as the mean ± *SD* of three independent experiments.
