# Supplementary figures and images for: A Novel Therapeutic Approach for Colorectal Cancer Stem Cells: Blocking the PI3K/Akt Signaling Axis With Caffeic Acid
Source: Front Cell Dev Biol. 2020 Dec 23;8:585987. doi: 10.3389/fcell.2020.585987 (PMC7785810; doi:10.3389/fcell.2020.585987)

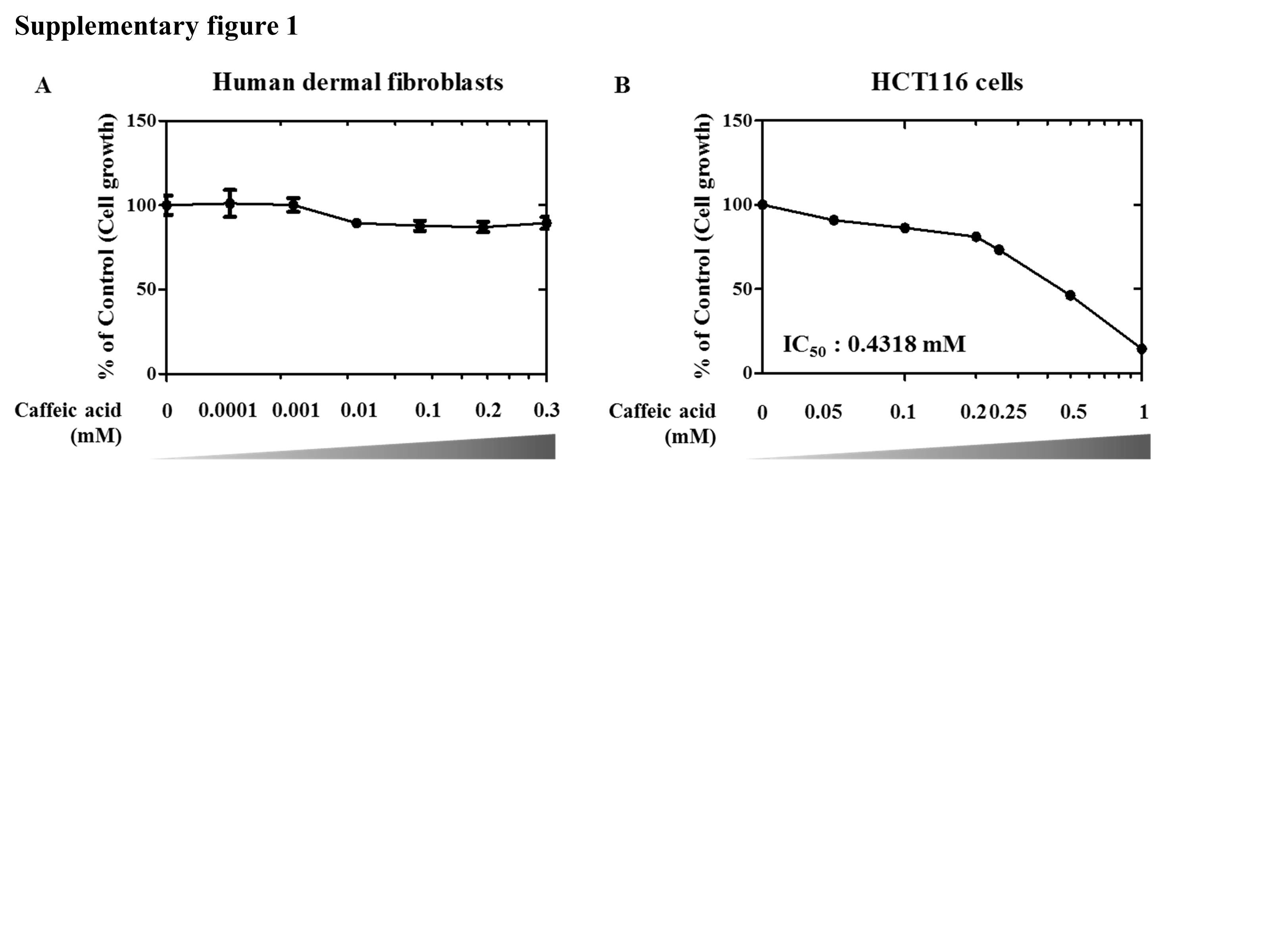

Supplement: Supplementary Figure 1 — IC50, the concentration that inhibits 50% of the proliferation of human dermal fibroblasts and HCT116 cells. The inhibition of cell viability by caffeic acid treatment for 48 h was determined by an MTT assay in human dermal fibroblasts (A) and HCT116 (B) cells. Cell viability (%) was calculated as a percent of the vehicle control. The results are presented as the mean ± SD of three independent experiments. [file Image_1.TIF]

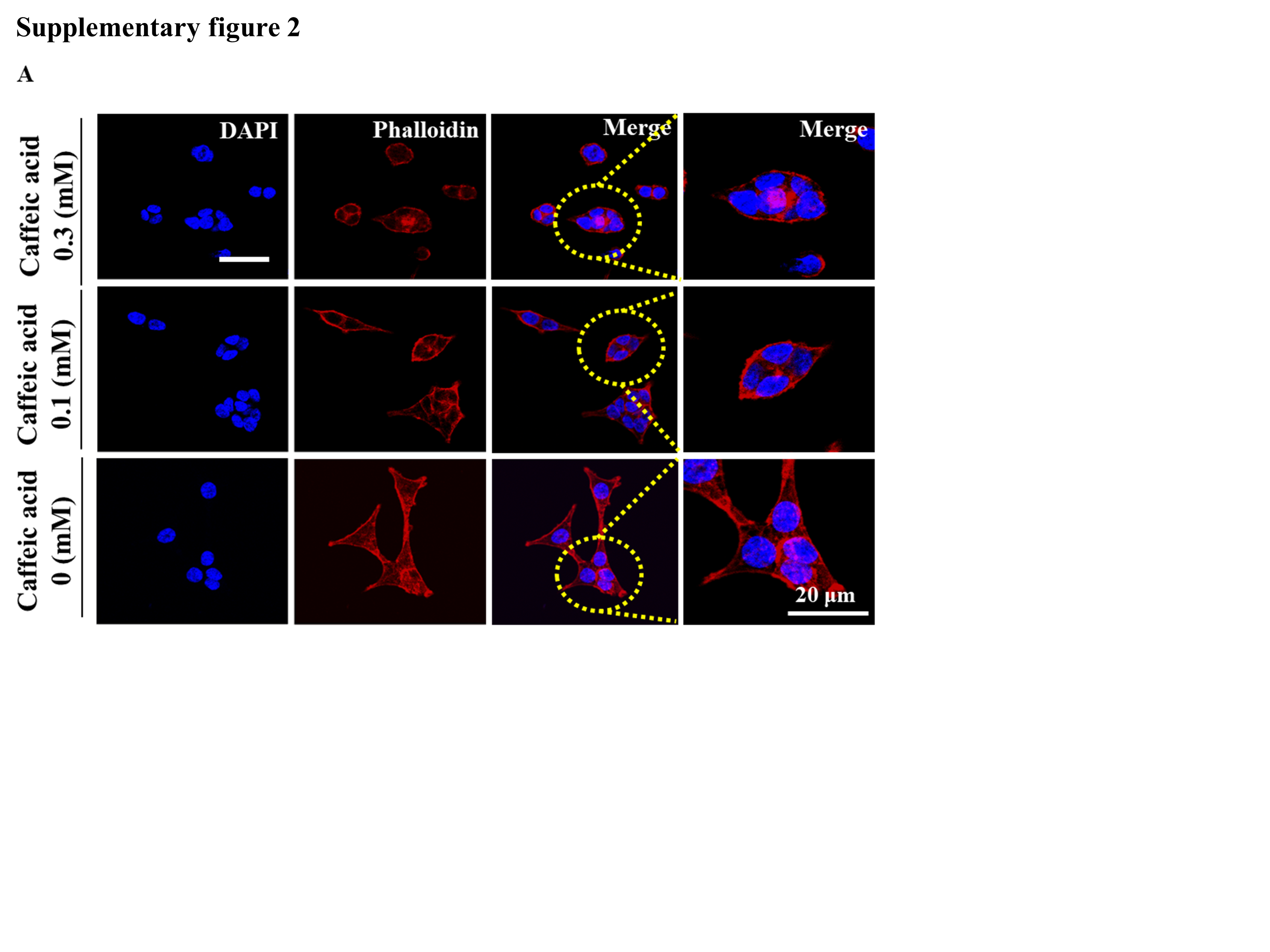

Supplement: Supplementary Figure 2 — The inhibitory effects of caffeic acid on the migratory capacity of HCT116 cells. Caffeic acid-induced actin filament disorganization and the morphological transition of HCT116 cells were visualized by staining the actin filaments with phalloidin. DAPI staining was used to label the nuclei within each field. [file Image_2.TIF]

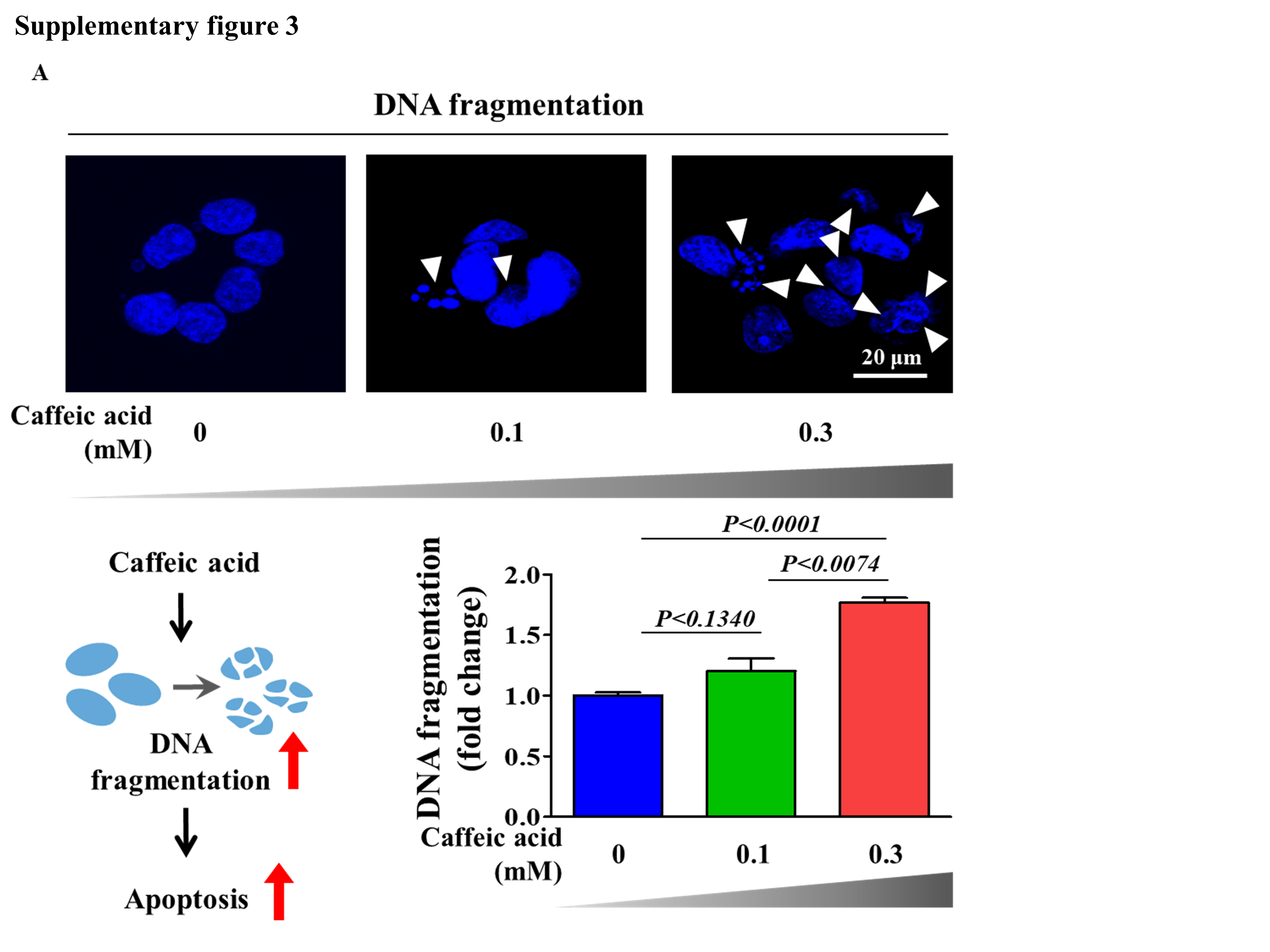

Supplement: Supplementary Figure 3 — The stimulatory effects of caffeic acid on the apoptotic DNA fragmentation in HCT116 cells. Caffeic acid-induced apoptotic DNA fragmentation and condensation were visualized using DAPI staining. The results are presented as the mean ± SD of three independent experiments. [file Image_3.TIF]
